# Randomised experimental evaluation of a social media campaign to promote COVID-19 vaccination in Nigeria

**DOI:** 10.7189/jogh.14.05018

**Published:** 2024-05-24

**Authors:** William D Evans, Jeffrey B Bingenheimer, Michael W Long, Khadidiatou Ndiaye, Dante Donati, Nandan M Rao, Selinam Akaba, Sohail Agha

**Affiliations:** 1 Milken Institute School of Public Health, The George Washington University, Washington D.C., USA; 2 School of Business, Columbia University, New York, USA; 3 Virtual Lab LLC, Corvallis, Oregon, USA; 4 Behavioral Insights Lab, Seattle, Washington, USA

## Abstract

**Background:**

The coronavirus disease 2019 (COVID-19) pandemic has challenged public health and behaviour change programmes, and has led to the development of innovative interventions and research. In low -and middle-income countries (LMICs) such as Nigeria, new strategies to promote vaccination, increase pro-vaccination social norms, and reduce vaccine hesitancy have been deployed through social media campaigns and evaluated using digital media platforms.

**Methods:**

We conducted two randomised experimental evaluations of social media content designed to promote COVID-19 vaccination and to complement research on a nationwide vaccination promotion campaign in Nigeria run in 2022. We conducted two studies in March and August 2022 among Nigerians drawn from 31 states that had not been targeted in the aforementioned nationwide campaign. We randomised the participants to either receive the pro-vaccination social media campaign or not and collected data at pre- and post-test time points to evaluate psychosocial predictors of vaccination and vaccination outcomes following the Theory of Change based on Diffusion of Innovations; the Social Norms Theory, and the Motivation, Opportunity, Ability (MOA) framework. Data were collected through a novel intervention delivery and data collection platform through social media.

**Results:**

We found that pro-vaccination social norms and vaccination rates increased, while vaccine hesitancy decreased among participants randomised to the social media intervention study arm.

**Conclusions:**

Social media campaigns are a promising approach to increasing vaccination at scale in LMICs, while social norms are an important factor in promoting vaccination, which is consistent with the Social Norms Theory. This study demonstrates the capability and potential of new social media-based data collection techniques. We describe implications for future vaccination campaigns and identify future research priorities in this area.

**Registration:**

Pan African Clinical Trial Registry: PACTR202310811597445.

The coronavirus disease 2019 (COVID-19) pandemic has been the greatest public health challenge of the 21st century. By the end of 2023, there had been approximately 772 000 000 confirmed cases and 7 000 000 deaths worldwide, according to the World Health Organization (WHO) [1]. The rapid development and deployment of vaccines in late 2020 and throughout the following years has been a tremendous success for the medical and public health communities, leading the WHO to declare the end of the COVID-19 public health emergency on 5 May 2023 [2]. However, they have also inevitably led to many challenges in communication and vaccination uptake, including misinformation and disinformation. This makes creating demand, promoting confidence in the safety and efficacy of COVID-19 vaccines, and understanding barriers to uptake critically important [3], especially among groups that are either uninformed, vaccine hesitant, or actively resistant to COVID-19 vaccination [4]. Crucial to this task is the understanding the factors that promote and reduce vaccine hesitancy, as well as the differences between population groups that lead to vaccine uptake and avoidance [5].

In low- and middle-income countries (LMICs), challenges in both the supply and demand for vaccination, including both COVID-19 and other routine vaccinations, are major issues that have been explored in recent social and behavioural research [6,7]. For example, in Nigeria, studies have reported public concerns about vaccine effectiveness, the severity of COVID-19, and distrust of government [8]. Reducing vaccine hesitancy in LMICs has been hypothesised to centre on the 5 Cs of vaccine hesitancy (confidence, complacency, convenience, calculation, and collective responsibility) [9]; social norms around vaccination [10,11]; and misinformation and disinformation, among other challenges [12]. Socio-demographic factors such as gender, gender equity, access to health care [13], education levels, socio-economic status, and endorsement by trusted actors such as health care providers (HCPs) have also been found to affect vaccine hesitancy and uptake in LMICs [14]. Given that previous studies showed vaccine hesitancy was a factor even among Nigerian HCPs, we sought to promote vaccination and pro-vaccine social norms among this important group [15].

Positive role models who promote a pro-vaccination norm, such as HCPs, business leaders, community and religious leaders, government officials, celebrities, and others are an important potential source of social influence to promote vaccination [16]. Demonstrating the benefits of vaccination and overcoming false and misleading information (eg, about negative health effects of vaccination, such as infertility) through influential messaging using widely available and used channels such as social media are potentially powerful avenues to increase vaccine uptake.

Our study is part of a wider project addressing the challenge of promoting COVID-19 vaccination in Nigeria, especially with influential actors such as HCPs. Within this project, a social media campaign [17] targeted social influencers (individuals who would be known and responded to positively by the intended audience). Within our study, we attempted to provide experimental evidence to support the efficacy of the campaign strategy under controlled conditions.

The use of a coherent Theory of Change (ToC), based on an established behavioural theory, is a key component of effective interventions to promote vaccination, as well as other public health programmes [18]. Previous evidence on the Nigeria social media campaign and the campaign’s ToC found that promoting positive social norms in favour of vaccination, reducing vaccine hesitancy, and promoting vaccination motivation, opportunity, and ability (MOA) would collectively lead to higher vaccination rates [17]. As in the previous study [17], the ToC for this campaign was based on the Diffusion of Innovations [19], Social Norms Theory [20,21], and the MOA framework [22]. The ToC specified primary and secondary outcomes in terms of COVID-19 vaccination, vaccine hesitancy, and the psychological and population-based processes by which changes in these outcomes occur, and was thus designed as the basis for a social media campaign in Nigeria using Facebook and Instagram, which have large followings in the country. The ToC hypothesised a set of mediating variables, including social norms, role modelling, and cultural beliefs, that are theoretically related to vaccine intentions and vaccination behaviour.

This study provides the first known randomised experimental evidence supporting a large-scale social media campaign to promote vaccination in a LMIC [23]. It complements and provides triangulating evidence in support of a previously published nationwide quasi-experimental study in Nigeria that demonstrated the impact of the campaign on vaccination rates and pro-vaccination social norms [17]. Here we conducted two randomised experiments to test the hypothesis that our previously observed effects of campaign social media messages on COVID-19 vaccination rates, vaccine hesitancy, and pro-vaccination social norms would be replicated. Confirming this hypothesis would provide additional support and triangulate findings from the national evaluation [23].

## METHODS

### Trial design

The current study was part of a larger evaluation of the impact of social media campaigns to promote COVID-19 vaccination, among health care workers and others in their social environment, in Nigeria, reported elsewhere [17]. The quasi-experimental nationwide evaluation employed mixed methods including a qualitative study of stakeholders, a cost-effectiveness study, and two quantitative studies. The quantitative studies included both a quasi-experimental, national evaluation [17] and, as reported here, an individual-level randomised controlled trial (RCT), conducted through social media-based surveys. Within the RCT study, we collected data from a Facebook Messenger based survey at two separate time points, March and April 2022, and again in August and September 2022. This study reports exclusively on the RCT findings.

We conducted two randomised experiments in which the intervention was delivered via Facebook using select content from the campaign within our previously-published quasi-experimental study. For both experiments, we recruited potential participants via advertisements on Facebook; they completed a baseline questionnaire and, if they were eligible, were randomised to a study arm using a simple randomisation algorithm programmed into the data collection platform. At this point, they were asked to complete a similar follow-up questionnaire one month later. The first experiment was carried out between March and April of 2022 and the second one between August and September of 2022. With this design we sought to provide evidence of the short-term effects of social media exposure on vaccine hesitancy, normative beliefs, and other outcomes from the campaign ToC. The first experiment had three arms: a zero-dose control arm, a low-dose treatment arm (defined as receiving a target of eight impressions of the social media content during the treatment period), and a high-dose treatment arm (defined as receiving a target of 16 impressions of the social media content during the treatment period). We used this approach to examine possible dosage and/or threshold effects of social media on vaccination outcomes. The second experiment had just two arms: control and treatment (defined as 16 impressions). In the first and second experiments, the treatment consisted of select content from the first or second phases of the campaign, respectively, being remarketed to the participants' Facebook/Instagram feeds at the specified dosage level.

### Participants

To be eligible, potential participants had to: reside in Nigeria, but not in one of the six states that received the campaign (to avoid contamination) in the previous quasi-experimental study; be at least 18 years of age at baseline/enrolment; be unvaccinated at baseline/enrolment; and belong to the ‘persuadable middle’ at baseline/enrolment. The latter three were assessed via self-report on the baseline questionnaire. Being in the ‘persuadable middle’ was operationalised as follows: if potential participants reported that they were not yet vaccinated on the baseline questionnaire, they were then asked ‘Would you take a COVID-19 vaccine that is approved for use in Nigeria if offered to you?’ We excluded those who responded ‘Definitely’ or ‘Definitely not’ and considered those who replied ‘Probably,’ ‘Probably not,’ ‘I may or I may not,’ or ‘Don’t know’ to be in the persuadable middle and therefore eligible for enrolment.

### Intervention

We report on the strategy used in the social media campaign elsewhere [17]. Briefly, it was designed and delivered by an independent team of creative designers and local organisations in Nigeria who curated social media content, customised it to specific geographical areas within the country, and delivered it on Facebook and Instagram. The local organisations included health care organisations, businesses, religious groups, and similar prominent leaders with a substantial, existing social media presence and network [17]. Content consisted of graphics, videos, and other social media materials such as graphic interface format (GIF) images. The campaign was run in two phases, from January to February 2022 and from May to September 2022, corresponding to the periods just after baseline; before the first follow-up; after the first follow-up; and just before the second follow-up.

Vaccine hesitancy occurs along a continuum between full acceptance (including high demand for vaccines) and outright refusal of some or all vaccines [24]. It is widely accepted that communications campaigns are best used to target people in the centre of a spectrum – the ‘persuadable middle.’ The campaign therefore attempted to reach HCPs and their communities who were unsure or delaying getting vaccinated.

Using the previously described ToC as a basis, the campaign designers iterated a series of social media content focussed on promoting pro-vaccination social norms and reducing vaccine hesitancy using social influencers (individuals such as local celebrities, other HCPs, religious and business leaders, and others who would be respected and trusted by the audience). The campaign’s ToC was premised on the idea that a social media campaign with high levels of reach and audience engagement would be able to reduce vaccine hesitancy, increase pro-vaccination social norms, and related attitudes and beliefs, thereby increasing COVID-19 vaccination uptake [17].

In this study, we used two criteria to select social media content to use in the randomised experiments: the posts with the highest Facebook/Instagram engagement statistics (greatest number of impressions and other activity such as likes and comments), where impressions were defined as the number of viewings of a social media post by a study participant [25]; and the campaign design team’s judgment as to which posts most closely aligned with the intended thematic content (the ToC) of the campaign. The campaign design team provided the data which we used to select the posts with the highest engagement statistics. We used content specific to the first campaign that ran in January to February 2022 in the first randomised experiment, and content specific to the second campaign from May to September 2022 in the second randomised experiment.

The George Washington University Institutional Research Board (IRB) and by the National Health Research Ethics Committee (NHREC) in Nigeria reviewed and approved the evaluation. We obtained consent from participants through consent statements provided in the social media chatbot (as described below). Participants who clicked to continue the survey after reading the statement consented to participate. We registered our trial with the Pan African Clinical Trial Registry (PACTR202310811597445) on 20 October 2023.

### Outcomes

We derived the measures for the questionnaire used in this study from the conceptual model of vaccine uptake and based them on the Diffusion of Innovations, the Social Norms Theory, and the MOA framework. Where available, we used existing, validated measures, including the 5 Cs model of vaccination acceptance [9]. We also adapted existing measures, such as social norms, to focus on the content of COVID-19 vaccination [26].

The focal independent variable in both experiments was the study arm. In the first experiment, this meant whether the participant was randomised to the no-dose (control) arm, the low-dose treatment arm, or the high-dose treatment arm. For most analyses, we operationalised this as two 0-or-1 indicator variables with the no-dose (control) arm. In the second experiment, there were only two arms – control and treatment – so we used a single 0-or-1 indicator variable signifying treatment arm.

The primary endpoint in this study was COVID-19 vaccination uptake. We assessed this using a single questionnaire item which read, ‘Have you received a COVID-19 vaccine?’ There were four response options: ‘Yes, a single-dose vaccine’; ‘Yes, the first dose of a two-dose regimen’; ‘Yes, both doses of a two-dose regimen’; and ‘No’. Because of changes over the course of the study in the availability of different types of COVID-19 vaccines in Nigeria, as well as recommendations for receiving boosters, we combined the first three responses into a single vaccinated category (coded 1), while those who answered ‘No’ were coded 0 for unvaccinated.

This study also had two secondary endpoints. The first was vaccine hesitancy, an index defined as the mean of coded responses to fifteen questionnaire items reflecting the 5 Cs framework [9]. Sample items read, ‘I am confident that COVID-19 vaccines are safe and effective,’ ‘Vaccination against COVID-19 is unnecessary,’ ‘Everyday stress prevents me from getting a COVID-19 vaccine,’ ‘When I think about getting vaccinated against COVID-19, I weigh the benefits and risks to make the best decision possible,’ and ‘When everyone is vaccinated against COVID-19, I don’t have to get vaccinated too.’ We used three questions for each of the five Cs: confidence, complacency, convenience, calculation, and collective responsibility, respectively. For each of these items, respondents were asked to select one of the following five response options: ‘Strongly agree,’ ‘Agree,’ ‘Neither agree nor disagree,’ ‘Disagree,’ and ‘Strongly disagree.’ We coded these 1 to 5 or 5 to 1, depending on whether, given the wording of the item, agreement reflected lower or higher levels of hesitancy. The resulting index had acceptable internal consistency (Cronbach’s a = 0.68 in the first experiment and 0.62 in the second). Because it is defined as the mean of component items, our vaccine hesitancy index has a plausible range of 1 to 5.

As in the previous nationwide quasi-experimental study, this study’s other secondary endpoint was pro-vaccination social norms, a scale score defined as the mean of coded responses to five questionnaire items [17]. The first two items read, ‘Your friends think it is important for everyone to get a COVID-19 vaccine’ and ‘Your family members think it is important for everyone to get a COVID-19 vaccine.’ The response options for each were ‘Strongly disagree,’ ‘Disagree,’ ‘Neither agree nor disagree,’ ‘Agree,’ and ‘Strongly agree,’ and we coded these as 1 to 5. The remaining three questionnaire items assessing social norms read, ‘Of the people close to you, what proportion of them would want you to get the COVID-19 vaccine?’ ‘How many people in Nigeria do you think will get the COVID-19 vaccine when it becomes available?’ and ‘How many people who work in health care in Nigeria do you think will get the COVID-19 vaccine when it becomes available?’ The response options for these were, ‘A few (1–20%),’ ‘Some (21–40%),’ ‘Many (41–60%),’ ‘Most (61–80%),’ and ‘Nearly all or all (81–100%).’ We coded these as 1 to 5, respectively. The resulting scale, defined as the mean of the component items, had a plausible range of 1 to 5 and a moderately high internal consistency (Cronbach’s α = 0.77 in the first experiment and 0.73 in the second). These measures were validated in the previous quasi-experimental study [17].

### Data collection

We selected survey data for this study and the overall evaluation of which it is a part on a social media-based research platform called Virtual Laboratory [27]. The survey instrument was designed to measure the previously mentioned outcomes, as well as key elements in the campaign ToC, described earlier.

Virtual Laboratory is an open-source software platform that uses targeted digital advertising to recruit a custom sample of respondents [28]. In this case, the study was stratified by health care worker status, and we sought to obtain 50% of our total sample in this group and the rest from general Nigerian population. To be eligible, targeted participants had to have a Facebook account through which they received recruitment advertising in their live feed promoting a study on COVID-19 vaccination. By clicking through the ad, potential participants then engaged with a chatbot that used Facebook Messenger to send reply chats. A series of Facebook Messenger chats then screened and offered them an opportunity to provide informed consent following the IRB-approved protocol. Eligible participants were offered an incentive of NGN 400 (about USD 1) in mobile phone credits, the same incentive scheme used in the quasi-experiment and other recent studies in Nigeria [17], to complete a questionnaire consisting of 40 items, each delivered as an individual Facebook Messenger message. Data were captured and stored in a secure Virtual Laboratory database.

Individuals who consented to participate were sent questionnaire items one at a time via the chatbot application. For most items, they were prompted to tap on their choice response to answer that item, after which they would receive the next item within a few seconds. This process was repeated until the end of the questionnaire. Responses were collected in an electronic database and exported as a .csv file for analysis.

### Data analysis

As in the previous quasi-experimental study [17], for our primary endpoint of COVID-19 vaccine uptake, we first obtained frequencies and percentages at each wave by study arm. Then, for vaccination status follow-up, we ran two linear regression models, one with the study arm indicators as the sole independent variables and the other with the following control variables: age group, gender, education, religion, and occupation, all of which were assessed at baseline. Religion and occupation were included to account for possible effects of support or opposition to vaccination in religious and workplace settings. All of these variables were included in analyses in the previous quasi-experimental study [17]. We used clustered standard errors (by state) for statistical inference in these models to account for the nesting of participants within states.

Similarly, for our two secondary endpoints of vaccine hesitancy and pro-vaccination social norms, we obtained descriptive statistics at each wave by study arm, and then used linear regression to test for differences between the study arms. For these outcomes, we included baseline levels of the outcome variable as an additional control variable in the adjusted models. As with our analysis of vaccination status, we used clustered standard errors to account for the nesting of participants within states.

Next, given attrition over the course of both experiments, we conducted an analysis of patterns of attrition. We created a 0-or-1 indicator variable for attrition between baseline and follow-up. We cross-tabulated each of these with our study arm indicator variables, age group, gender, education, religion, and occupation. We also created categorical versions of our baseline vaccine hesitancy index and social norms variables, with scores between 1 and 2 categorised as ‘lowest,’ scores between 2 and 3 categorised as ‘low,’ scores between 3 and 4 categorised as ‘high,’ and scores between 4 and 5 categorised as ‘highest.’ We cross-tabulated these with our attrition indicators, just as we did for the study arm and the socio-demographic variables. For each potential determinant of attrition, we also estimated a logistic regression model (again using clustered standard errors) to determine whether that variable’s association with attrition was statistically significant. We also tested for differential attrition across arms using logistic regression models with the study arm indicator variables, one of the baseline variables (age, gender), and the study arm indicator by baseline variable interaction terms. We used likelihood ratio tests of nested models (with vs without the interaction terms) to test whether the pattern of attrition was differential for each baseline variable.

Finally, as robustness checks, we conducted two alternative versions of our main analyses. In the first, we used carry-forward imputation for missing values of vaccination status, vaccine hesitancy, and pro-vaccination social norms at first and second follow-ups. Because being vaccinated at enrolment was an exclusion criterion, this meant that everyone who was lost to follow-up was assumed to still be unvaccinated at those follow-up time points in these analyses. We repeated the primary analyses using the data set with carry-forward imputation for missing values. This approach, which involves carrying forward values of outcome variables from earlier waves, has the advantages of simplicity and transparency, and tends to be statistically conservative. Next, we used multiple imputation by chained equations to create 50 completed data sets to make full use of the set of non-missing values and account for statistical uncertainty in the imputations [29]. Input variables for the imputations included all baseline demographic variables, as well as the baseline vaccine hesitancy index and pro-vaccination social norms scale. We then carried out analyses analogous to our primary ones using these imputed data sets. All data management and analysis were carried out in State 17. Clustered standard errors were obtained by adding the option ‘vce(cluster state)’ to all linear and logistic regression commands. For the multiple imputations and the analyses of the imputed data sets we used Stata, version 2020 (StataCorp LLC, College Station, TX, USA) and the ‘mi impute chained’ command and regression commands with the ‘mi estimate’ prefix.

## RESULTS

**Figure 1:**
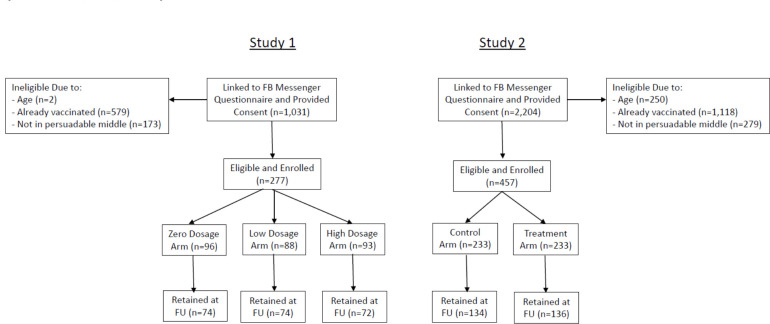
CONSORT Diagram for studies 1 and 2.

A total of 1031 potential participants linked to the Facebook Messenger questionnaire in study 1 and 2204 linked to the questionnaire in study 2 began answering screening questions. A total of 277 were eligible and enrolled in study 1, and 457 were eligible and enrolled in study 2, with already having received a vaccination against COVID-19 accounting for the majority of the exclusions, followed by not being in the ‘persuadable middle’ in terms of intention to get vaccinated; missing baseline variables; being below age 18; and having a duplicate ID number (which occurred when someone from a single Facebook account linked to the questionnaire more than once). After enrolment, participants were randomised to the study arm. At follow-up, 220 participants were retained in study 1 and 270 in study 2 (**Figure 1**).

The sample was young, with over 84% being under the age of 40 years in study 1 and 77% in study 2 (**Table 1**). A small majority were women, and more than half had earned a bachelor’s degree or higher. The most common religious affiliation was non-Catholic Christian, followed by Roman Catholic in study 1 and by Muslim in study 2, and others. About 58% were not HCPs in study 1 and 45% were not HCPs in study 2; those who were HCPs were distributed across various occupations. We observed some evident differences in the socio-demographic composition of participants across the study arms.

**Table 1:** Sociodemographic composition of study 1 and study 2 baseline samples by treatment assignment, presented as n (%)

	Study 1				Study 2		
	**None (n = 96)**	**Low (n = 88)**	**High (n = 93)**	**Total (n = 277)**	**Control (n = 233)**	**Treatment (n = 224)**	**Total (n = 457)**
**Age in years**							
18–29	48 (50.0)	45 (51.1)	41 (44.1)	134 (48.4)	95 (40.8)	96 (42.9)	191 (41.8)
30–39	36 (37.5)	31 (35.2)	33 (35.5)	100 (36.1)	86 (36.9)	77 (34.4)	163 (35.7)
40–49	7 (7.3)	11 (12.5)	16 (17.2)	34 (12.3)	37 (15.9)	36 (16.1)	73 (16.0)
50–59	3 (3.1)	0 (0.0)	3 (3.2)	6 (2.2)	10 (4.3)	7 (3.1)	17 (3.7)
60+	2 (2.1)	1 (1.1)	0 (0.0)	3 (1.1)	5 (2.2)	8 (3.6)	13 (2.8)
**Gender**							
Man	47 (49.0)	32 (36.4)	44 (47.3)	123 (44.4)	104 (44.6)	93 (41.5)	197 (43.1)
Woman	48 (50.0)	55 (62.5)	49 (52.7)	152 (54.9)	129 (55.4)	125 (55.8)	254 (55.6)
Prefer not to say	1 (1.0)	1 (1.1)	0 (0.0)	2 (0.7)	0 (0.0)	6 (2.7)	6 (1.3)
**Education**							
Secondary	12 (12.5)	13 (14.8)	9 (9.7)	34 (12.3)	34 (14.6)	33 (14.7)	67 (14.7)
Diploma	14 (14.6)	18 (20.5)	16 (17.2)	48 (17.3)	43 (18.5)	33 (14.7)	76 (16.6)
Bachelors	53 (55.2)	49 (55.7)	53 (57.0)	155 (56.0)	117 (50.2)	126 (56.3)	243 (53.2)
Masters	10 (10.4)	4 (4.6)	7 (7.5)	21 (7.6)	22 (9.4)	25 (11.2)	47 (10.3)
Doctorate	0 (0.0)	0 (0.0)	2 (2.2)	2 (0.7)	5 (2.2)	1 (0.5)	6 (1.3)
Other	7 (7.3)	4 (4.6)	6 (6.5)	17 (6.1)	12 (5.2)	6 (2.7)	18 (3.9)
**Religion**							
Other Christian	68 (70.8)	62 (70.5)	60 (64.5)	190 (68.6)	158 (67.8)	151 (67.4)	309 (67.6)
Catholic	11 (11.5)	15 (17.1)	17 (18.3)	43 (15.5)	28 (12.0)	32 (14.3)	60 (13.1)
Muslim	16 (16.7)	8 (9.1)	13 (14.0)	37 (13.4)	36 (15.5)	39 (17.4)	75 (16.4)
Other	1(1.0)	3 (3.4)	3 (3.2)	7 (2.5)	11 (4.7)	2 (0.9)	13 (2.8)
**Employment/occupation**							
Not in the health sector	36(37.5)	39 (44.3)	41 (44.1)	116 (41.9)	128 (54.9)	124 (55.4)	252 (55.1)
Nurse/midwife	3 (3.1)	10 (11.4)	10 (10.8)	23 (8.3)	16 (6.9)	22 (9.8)	38 (8.3)
Other public health	7 (7.3)	8 (9.1)	5 (5.4)	20 (7.2)	17 (7.3)	14 (6.3)	31 (6.8)
Laboratory staff	9 (9.4)	4 (4.6)	5 (5.4)	18 (6.5)	9 (3.9)	7 (3.1)	16 (3.5)
Community health worker	3 (3.1)	2 (2.3)	3 (3.2)	8 (2.9)	5 (2.2)	5 (2.2)	10 (2.2)
Medical doctor	5 (5.2)	1 (1.1)	0 (0.0)	6 (2.2)	2 (0.9)	1 (0.5)	3 (0.7)
PPMV/Chemist	3 (3.1)	0 (0.0)	1 (1.1)	4 (1.4)	2 (0.9)	2 (0.9)	4 (0.9)
Pharmacist	0 (0.0)	1 (1.1)	1 (1.1)	2 (0.7)	5 (2.2)	2 (0.9)	7 (1.5)
Unemployed	30 (31.3)	23 (26.1)	27 (29.0)	80 (28.9)	49 (21.0)	47 (21.0)	96(21.0)

PPMV – patent and proprietary medicine vendors

In view of the primary and secondary endpoints in study 1 (**Table 2**), the crude and adjusted differences in vaccination rates between the low-dose and zero-dose (control) arms were 0.0 and 0.6 percentage points, respectively, while the crude and adjusted differences between the high-dose and zero-dose control arms were 1.6 and 1.3 percentage points, respectively. None of these differences were statistically distinguishable from the null. Vaccine hesitancy scale scores decreased significantly between the low treatment and control condition in the adjusted models (−0.15, *P* < 0.030) and approached a significant decrease between the high treatment and control condition in the adjusted models (−0.12, *P* < 0.074). Scores on the pro-vaccination social norms scale increased significantly between the low treatment and control condition in the adjusted models (0.22, *P* < 0.044) and between the high treatment and control condition in the adjusted models (0.23, *P* < 0.023).

**Table 2:** Levels of vaccination uptake, vaccine hesitancy, and pro-vaccination norms, and crude and adjusted differences at follow-up, study 1

	Vaccinated, % (n/N)			Vaccine hesitancy scale, x̄ (SD)			Pro-vaccination social norms scale, x̄ (SD)		
**Levels**	**None**	**Low**	**High**	**None**	**Low**	**High**	**None**	**Low**	**High**
Baseline	0.0 (0/96)	0.0 (0/88)	0.0 (0/93)	2.84 (0.42)	2.89 (0.45)	2.90 (0.45)	3.00 (0.71)	2.88 (0.87)	2.80 (0.89)
Follow-up	6.8 (5/74)	6.8 (5/74)	8.3 (6/72)	2.93 (0.46)	2.79 (0.37)	2.86 (0.39)	2.99 (0.81)	3.08 (0.75)	3.04 (0.84)
Differences at follow-up*									
Low vs none, crude	0.0 (0.999)			−0.13 (0.113)			0.08 (0.566)		
Low vs none, adjusted	0.6 (0.888)			−0.15 (0.030)			0.22 (0.044)		
High vs none, crude	1.6 (0.660)			−0.06 (0.413)			0.05 (0.673)		
High vs none, adjusted	1.3 (0.731)			−0.12 (0.074)			0.23 (0.023)		

SD – standard deviation, x̄ – mean

*Presented as estimate (*P*-value).

For the main findings on the primary and secondary endpoints at the follow-up questionnaire in study 2 (**Table 3**), the crude and adjusted increases in vaccination rates at follow-up in percentage point terms were 6.5 and 6.0; these approached, but did not reach statistical significance (*P* < 0.057 and *P* < 0.085, respectively). Vaccine hesitancy scale and pro-vaccination social norms scale scores were not statistically distinguishable from the null in both the crude and adjusted models.

**Table 3:** Levels of vaccination uptake, vaccine hesitancy, and pro-vaccination norms, and crude and adjusted differences at follow-up, study 2

	Vaccinated, % (n/N)		Vaccine hesitancy scale, x̄ (SD)		Pro-vaccination social norms scale, x̄ (SD)	
**Levels**	**Treatment**	**Comparison**	**Treatment**	**Comparison**	**Treatment**	**Comparison**
Baseline	0.0 (0/224)	0.0 (0/233)	2.92 (0.42)	2.88 (0.38)	2.90 (0.79)	2.91 (0.71)
Follow-Up	16.2 (22/136)	9.7 (13/134)	2.89 (0.43)	2.90 (0.37)	3.07 (0.82)	2.98 (0.71)
						
Differences at follow-up (treatment – comparison)*						
Crude	6.5 (0.057)		−0.01 (0.757)		0.09 (0.369)	
Adjusted	6.0 (0.085)		−0.00 (0.928)		0.05 (0.419)	

SD – standard deviation, x̄ – mean

*Presented as estimate (*P*-value).

In view of the results of our attrition and sensitivity analyses for study 1, where 20.6% of enrolled participants were lost to follow-up (Table S1 in the **Online Supplementary Document**), only one variable was statistically significantly associated with attrition: older participants were more likely than younger participants to be lost to follow-up. Analyses with study-arm-by-baseline-variable interactions provided no evidence for differential attrition across the study arms. Results of our sensitivity analyses using carry-forward and multiple imputation by chained equations are presented in Tables S2 and S3 in the **Online Supplementary Document**, respectively. Point estimates from these analyses are all similar to those presented in **Table 2**, but some differences that were statistically significant at the 0.05 level in our main analyses became non-significant in these sensitivity analyses. For study 2, where loss-to-follow-up was higher at 40.9%, two variables, age and education, were statistically significantly associated with attrition (Table S4 in the **Online Supplementary Document**). As in study 1, however, there was no evidence of differential attrition across study arms in the analyses with arm-by-baseline-variable interactions. As shown in Tables S5 and S6 in the **Online Supplementary Document**, analyses using carry-forward and multiple imputation by chained equations yielded very similar point estimates to those shown in **Table 3**. Effects on vaccine uptake, which did not achieve statistical significance in our main analyses, became significant in the sensitivity analyses using carry-forward imputation, but not in the one using multiple imputation, while effects on vaccine hesitancy and pro-vaccination social norms remained non-significant.

## DISCUSSION

This study aimed to provide the first-ever RCT evidence on the effects of a large-scale social media campaign to promote COVID-19 vaccination in a LMIC. Given its novelty and the importance of digital health and vaccination promotion research, especially in LMICs, it can potentially improve the evidence base in the field [30]. Within the study, we tested a set of hypotheses based on a grounded ToC, which has been absent from many previous social media campaigns in LMICs [31].

Overall, this study partially confirmed findings from the previous, nationwide quasi-experimental evaluation of the Nigerian COVID-19 vaccination social media campaign. We found evidence that pro-vaccination social norms increased among the treatment group in study 1, confirming our hypothesis that the nationwide evaluation findings would be replicated in a randomised experiment. We also found evidence that vaccine hesitancy was reduced among the treatment group in study 1, which was not observed in the nationwide study. Finally, we found some directional evidence approaching, but not reaching statistical significance, for an increase in COVID-19 vaccination rates among the treatment group in study 2.

The magnitude of the findings was similar in these two experimental studies to those observed in the nationwide evaluation. Overall, these experiments provide further support for the social media campaign's effectiveness in promoting COVID-19 vaccination and th erelated psychosocial predictors of vaccination, with noted limitations.

While this study is consistent with and provides experimental evidence to support the previous nationwide findings, these results differ in some respects from that larger evaluation. In particular, the observed reduction in vaccine hesitancy in study 1 is of interest, and it provides support for the hypothesis that social media can affect hesitancy. The lack of consistent results for psychosocial predictors across both study 1 and study 2 may be attributable to loss-to-follow-up in the two studies, and future research with larger sample sizes and greater statistical power should be conducted to examine this hypothesis. The vaccination findings, while approaching but not reaching statistical significance, are consistent with the nationwide study. We note that these findings are broadly consistent with the campaign’s ToC, which predicted that vaccination rates would be affected by reduced vaccine hesitancy and increased pro-vaccination social norms.

The finding that pro-vaccination social norms may be impacted by a COVID-19 vaccination social media campaign is noteworthy in terms of the mechanism of change. As discussed in our previous study [17], future research on social media vaccination campaigns should explore the mechanisms by which social norms are changed and how they influence vaccination outcomes. The reach and scale of social media may create a widespread sense that vaccination is what most of one’s peers are doing and that it is widely socially accepted [12]. The mechanisms by which social media influences vaccination social norms also deserve further experimental research. In particular, HCPs served as pro-vaccination role models in the social media content, which should be further explored as a mechanism for reducing hesitancy and improving vaccination social norms.

In terms of practical significance, this study confirms the findings from the previous quasi-experiment [17] that social media campaigns can impact vaccination outcomes, including their psychosocial precursors. However, the relatively small size of these effects in absolute terms, as well as the marginal and non-significant observed effects, should be considered in designing future interventions and studies.

### Recommendations

Recommendations for future research also include employing RCT designs to evaluate social media health promotion in other settings and topics. Other randomised designs, such as cluster randomisation, should be employed in future studies on social media programmes for health promotion [32]. The availability of social media-based data collection and intervention delivery platforms such as Virtual Laboratory creates an opportunity for innovation and naturalistic RCT study designs that may be applied across the range of health promotion and disease prevention domains.

Further development and evaluation of outcomes measures used here, including vaccine hesitancy and social norms, should also be done in the future. Finally, evaluation of the campaign theory of change should be conducted through formal mediation analysis (structural equation modelling).

### Limitations

Limitations of the current research include potential contamination of participants in the non-treatment states from the nationwide campaign from which our sample was drawn. While we did not see evidence of such contamination and no evidence of the campaign reaching our study participants, travel to treatment states, sharing of content on social media, and other possible sources of contamination cannot be entirely ruled out. Additionally, we only employed short-term follow-ups that were shorter than the nationwide quasi-experimental evaluation. Long-term follow-up would have provided further and stronger evidence of the intervention’s effectiveness. Finally, we experienced significant loss-to-follow-up, which may have affected our findings, especially for vaccination. It is also possible that there was reporting bias due to sampling only social media users. Finally, the approach of restricting our analysis to the persuadable middle sub-sample may require further validation. These findings may have limited generalizability due to the Nigerian context. Future studies using social media-based data collection should make additional efforts to reach out to study participants, include other geographical locations, provide additional forms of incentives to remain in the study, and reduce loss-to-follow-up to maximise statistical power and reduce potential bias [33].

Finally, this evaluation contributes to both the knowledge about digital interventions in LMICs (specifically in Nigeria) and interventions for HCPs in LMICs. The health care sector is growing rapidly along with the population in LMICs, especially in sub-Saharan Africa [34]. Best practices to improve care include a focus on HCP’s behaviour, especially in view of their role in promoting population health through role modelling of healthy behaviours such as vaccination. This project provides an example of best practice in promoting healthy behaviours among HCPs in a LMIC setting.

## CONCLUSIONS

In an RCT, we confirmed findings from the nationwide evaluation showing that the social media campaign had generally positive effects of COVID-19 vaccination and psychosocial predictors of vaccination. We also found evidence that vaccine hesitancy may be influenced by social media messages. Future research should build on the RCT methods using social media platforms demonstrated here to evaluate other vaccination and a broader range of health promotion and disease prevention interventions [35].

## Additional material


Online Supplementary Document
