# Supplementary material for: Randomised experimental evaluation of a social media campaign to promote COVID-19 vaccination in Nigeria
Source: J Glob Health. 2024 May 24;14:05018. doi: 10.7189/jogh.14.05018 (PMC11112529; doi:10.7189/jogh.14.05018)
Supplement: Online Supplementary Document [file jogh-14-05018-s001.pdf]

Table S1. Patterns of Loss-to-Follow-Up (LTFU) Between Baseline and Follow-up, Study 1

	N at Baseline	LTFU %	OR	p-value	Omnibus p-value	Interaction p-value
Exposure Level						
None	96	22.9	1.00	Ref	0.472	
Low	88	15.9	0.64	0.300		
High	93	22.6	0.98	0.953		
Age Group						
18 to 29 years	134	13.4	1.00	Ref	0.002	0.934
30 to 39 years	100	24.0	2.04	0.053		
40+ years	23	34.9	3.45	0.001		
Gender						
Male	123	23.6	1.00	Ref	0.250	0.517
Female	152	18.4	0.73	0.250		
Prefer Not to Say	2	0.0	--	--		
Education						
Secondary	34	20.6	1.00	Ref	0719	0.404
Diploma	48	14.6	0.66	0.456		
Bachelors	155	21.9	1.08	0.855		
Higher/Other	40	22.5	1.12	0.835		
Employment						
Health Sector	81	17.3	1.00	Ref	0.367	0.999
Not Health Sector	116	19.8	1.18	0.663		
Unemployed	80	25.0	1.60	0.196		
Religion						
Catholic	43	25.6	1.00	Ref	0.327	0.198
Muslim/Other	44	13.6	0.46	0.139		
Other Christian	190	21.1	0.78	0.508		
Baseline Hesitancy						
Low	166	20.5	1.00	Ref	0.957	0.859
High	111	20.7	1.01	0.957		
Baseline Social Norms						
Lowest	30	23.3	1.00	Ref	0293	0.643
Low	114	24.6	1.07	0.879		
High	103	17.5	0.70	0.440		
Highest	30	13.3	0.51	0.353		

Table S2. First Robustness Check Results: Levels of Vaccination Uptake, Vaccine Hesitancy, and Pro-Vaccination Norms, and Crude and Adjusted Differences at Follow-Up with Simple Carry-Forward Imputation, Study 1

	Vaccinated Percent and (Count)			Vaccine Hesitancy Scale Mean and (SD)			Pro-Vaccination Social Norms Scale Mean and (SD)		
Levels	None	Low	High	None	Low	High	None	Low	High
Baseline	0.0% (0/96)	0.0% (0/88)	0.0% (0/93)	2.84 (0.42)	2.89 (0.45)	2.90 (0.45)	3.00 (0.71)	2.88 (0.87)	2.80 (0.89)
Follow-Up	5.2% (5/96)	5.7% (5/88)	6.5% (6/93)	2.92 (0.46)	2.84 (0.40)	2.85 (0.41)	2.96 (0.78)	3.02 (0.76)	2.97 (0.84)
Differences at Follow-Up		Estimate	(p-value)		Estimate	(p-value)		Estimate	(p-value)
Low vs None, Crude		0.4	(0.888)		-0.08	(0.289)		0.06	(0.585)
Low vs None, Adjusted		1.2	(0.742)		-0.12	(0.036)		0.18	(0.027)
High vs None, Crude		1.2	(0.651)		-0.07	(0.271)		0.00	(0.968)
High vs None, Adjusted		2.2	(0.436)		-0.11	(0.024)		0.17	(0.026)

Supplemental Table C. Second Robustness Check Results: Levels of Vaccination Uptake, Vaccine Hesitancy, and Pro-Vaccination Norms, and Crude and Adjusted Differences at Follow-Up with Multiple Model-Based Imputation via Chained Equations, Study 1

	Vaccinated Percent and (Count)			Vaccine Hesitancy Scale Mean and (SD)			Pro-Vaccination Social Norms Scale Mean and (SD)		
Levels	None	Low	High	None	Low	High	None	Low	High
Baseline	0.0% (0/96)	0.0% (0/88)	0.0% (0/93)	2.84 (0.42)	2.89 (0.45)	2.90 (0.45)	3.00 (0.71)	2.88 (0.87)	2.80 (0.89)
Follow-Up	8.9% (8/96)	8.0% (7/88)	9.6% (9/93)	2.91 (0.46)	2.83 (0.38)	2.87 (0.40)	3.00 (0.83)	3.04 (0.75)	3.01 (0.84)
Differences at Follow-Up		Estimate	(p-value)		Estimate	(p-value)		Estimate	(p-value)
Low vs None, Crude		-0.8	(0.862)		-0.08	(0.262)		0.04	(0.712)
Low vs None, Adjusted		-0.4	(0.933)		-0.11	(0.053)		0.17	(0.088)
High vs None, Crude		0.8	(0.856)		-0.04	(0.526)		0.01	(0.910)
High vs None, Adjusted		1.8	(0.683)		-0.10	(0.116)		0.17	(0.105)

Table S3. Patterns of Loss-to-Follow-Up (LTFU) Between Baseline and Follow-up, Study 2

	N at Baseline	LTFU %	OR	p-value	Omnibus p-value	Interaction p-value
Study Condition						
Control	233	42.5	1.00	Ref	0.507	
Treatment	224	39.3	0.88	0.507		
Age Group						
18 to 29 years	191	36.7	1.00	Ref	0.000	0.357
30 to 39 years	163	36.2	0.98	0.938		
40 to 49 years	73	57.5	2.34	0.000		
50+	30	53.3	1.98	0.063		
Gender						
Male	197	39.6	1.00	Ref	0.706	0.415
Female	254	42.1	1.11	0.554		
Prefer Not to Say	6	33.3	0.76	0.694		
Education						
Secondary	67	40.3	1.00	Ref	0.000	0.383
Diploma	76	43.4	1.14	0.714		
Bachelors	243	35.4	0.81	0.512		
Masters	47	61.7	2.39	0.088		
Doctorate	6	83.3	7.41	0.103		
Other	18	38.9	0.94	0.914		
Employment						
Doctor/Nurse/Midwife	41	46.3	1.00	Ref	0.481	0.057
CHW/Other Public Health	27	25.9	0.41	0.109		
Pharmacist/Lab/PPMV/Chemist	41	46.3	1.00	1.000		
Not a Health Sector Worker	252	40.9	0.80	0.488		
Unemployed	96	40.6	0.79	0.342		
Religion						
Catholic	60	43.3	1.00	Ref	0.950	0.923
Muslim	75	38.7	0.82	0.585		
Other Christian	309	41.1	0.91	0.754		
Traditionalist/Other	13	38.5	0.82	0.738		
Baseline Hesitancy						
Low	263	41.1	1.00	Ref	0.646	0.923
Medium	187	40.1	0.96	0.855		
High	7	57.1	1.91	0.431		
Baseline Social Norms						
Lowest	36	44.4	1.00	Ref	0.972	0.921
Low	203	39.9	0.83	0.656		
High	165	40.6	0.85	0.709		
Highest	53	43.4	0.96	0.923		

Table S4. First Robustness Check Results: Levels of Vaccination Uptake, Vaccine Hesitancy, and Pro-Vaccination Norms, and Crude and Adjusted Differences at Follow-Up with Simple Carry-Forward Imputation, Study 2

	Vaccinated Percent and (Count)		Vaccine Hesitancy Scale Mean and (SD)		Pro-Vaccination Social Norms Scale Mean and (SD)	
Levels	Treatment	Comparison	Treatment	Comparison	Treatment	Comparison
Baseline	0.0% (0/224)	0.0% (0/233)	2.92 (0.42)	2.88 (0.38)	2.90 (0.79)	2.91 (0.71)
Follow-Up	9.8% (22/224)	5.6% (13/233)	2.90 (0.42)	2.89 (0.37)	2.98 (0.84)	2.94 (0.73)
Differences at Follow-Up (T – C)	Estimate	(p-value)	Estimate	(p-value)	Estimate	(p-value)
Crude	4.2	(0.041)	0.00	(0.911)	0.05	(0.523)
Adjusted	4.2	(0.062)	-0.01	(0.790)	0.06	(0.120)

Supplemental Table F. Second Robustness Check Results: Levels of Vaccination Uptake, Vaccine Hesitancy, and Pro-Vaccination Norms, and Crude and Adjusted Differences at Follow-Up with Multiple Model-Based Imputation via Chained Equations, Study 2

	Vaccinated Percent and (Count)		Vaccine Hesitancy Scale Mean and (SD)		Pro-Vaccination Social Norms Scale Mean and (SD)	
Levels	Treatment	Comparison	Treatment	Comparison	Treatment	Comparison
Baseline	0.0% (0/224)	0.0% (0/233)	2.92 (0.42)	2.88 (0.38)	2.90 (0.79)	2.91 (0.71)
Follow-Up	17.2% (39/224)	13.0% (30/233)	2.89 (0.43)	2.91 (0.40)	3.01 (0.85)	2.99 (0.78)
Differences at Follow-Up (T – C)	Estimate	(p-value)	Estimate	(p-value)	Estimate	(p-value)
Crude	4.2	(0.269)	-0.02	(0.659)	0.02	(0.845)
Adjusted	4.2	(0.269)	-0.01	(0.889)	0.02	(0.737)
